# Effects of garlic supplementation on energy status of pre-partum Mahabadi goats

**Published:** 2014

**Authors:** Rasoul Pirmohammadi, Ehsan Anassori, Zahra Zakeri, Mortaza Tahmouzi

**Affiliations:** 1 *Department of Animal Science, Faculty of Agriculture, Urmia University, Urmia, Iran; *; 2 *Department of Clinical Sciences, Faculty of Veterinary Medicine, Urmia University, Urmia, Iran.*

**Keywords:** β-Hydroxybutyrate, Glucose, Mahabadi goat, Non-esterified fatty acids, Raw garlic

## Abstract

To evaluate the effects of garlic on some blood metabolites in pre-partum dairy goats, the ration was supplemented with raw garlic at the doses of 0, 30, 50 and 70 g kg^-1^ of Dry matter (DM) in eight pregnant Mahabadi breed goats (59 ± 1 kg initial live weight) in a replicated Latin square design during the last two months of pregnancy. Each experimental period lasted 14 days with the first 12 days used for diet adaptation and two days of data collection with a 3-days wash-out period between treatments. The results revealed a beneficial increasing effect of garlic (at the level of 70 g kg^-1^ of DM) on serum glucose concentration (*p* < 0.05). No effects of garlic supplementation on blood non-esterified fatty acids (NEFA), β-hydroxybutyrate (BHB), total triglycerides, total protein, and albumin concentration were observed, except for serum cholesterol concentration, which was reduced (*p *< 0.05) with 70 g kg^-1^ of DM of garlic supplementation. Dry matter intake was the same between the different treatment groups and throughout the trial period. Concerning the blood indicators of negative energy balance, no significant effects were found for NEFA and BHB in pre-partum goats however, serum glucose was improved significantly, which showed that garlic supplementation may improve the efficiency of feed utilization. In conclusion, garlic, as feed additives in ruminant nutrition, holds promise for improving feed efficiency and controlling the negative energy balance.

## Introduction

Nutritional requirements during the last third of gestation are very high in small ruminants due to the high prolificacy of these animals. In goats, the conceptus acquires 80.00% of its birth weight in the last two months of gestation.^1^ This means that a pregnant goat's daily energy requirements just before parturition can be increased by 2.5 times compared to the early gestation for the same animal.^2^ In order to cover the requirements for fetal growth, the goat must increase the feed intake or mobilize its reserves. Feed intake can be low at the end of gestation due to compression of the rumen by the fetuses which reduces the rate at which the products of fermentation-synthesis are absorbed.^3^ An extreme negative energy balance can result in fatal consequences, such as pregnancy toxemia, in pre-parturient ewes and goats.^4^ The precipitating cause for these consequences is the increased energy demand of the conceptus in the latter part of pregnancy. The most important etiological factor in pregnancy toxemia is a decline in the plane of nutrition during the last 4 to 6 weeks of pregnancy. This is the period when fetal growth is rapid and the demands for energy is markedly increased.^5^

Any attempt to diminish the negative effect of feed intake depression and negative energy balance during the transition period should improve the nutritional and metabolic status and also well-being of dairy goats. In recent years, aromatic plants and their extracts have received increased attention as potential alternatives to growth promoters. Garlic (*Allium sativum*) has been used as spice and folk medicine since antiquity.^6^ Bioactive components of garlic, including several sulfur-containing compounds such as alliin, diallylsulfides and allicin, may partly account for some effects of garlic.^7^ Effects of some these secondary compounds have already been demonstrated on rumen manipulation (e.g. defaunation, decreased methane production, decreased ruminal degradation of dietary proteins, reducing the proportion of acetate and increasing that of propionate in a manner similar to monensin).^8^^-^^9^ The hypothesis addressed in this paper was that dietary addition of raw garlic would lead to improvements in feed utilization and in energy related blood metabolites.

## Materials and Methods

**Experimental design, animal management and diets. **Eight pregnant Mahabadi breed, a native breed, goats carrying twins with the age of 4 years, and body weight of 59 ± 1 kg were selected according to probable kidding date from previously estrous synchronized herd. The twining status was diagnosed with ultrasonography (Model 100 LC VET scanner; Pie Medical, Maastricht, The Netherland). The animals were randomly assigned to a replicated 4×4 Latin square design with 12 days of adaptation and two days of sampling periods with a 3-days wash-out period between treatments. The study was started and completed in the last two months of pregnancy. At day 80 ± 1 of pregnancy, the goats were divided into four groups, housed indoor in individual pen (5 m^2^) and were fed daily a diet designed to cover 16.10 MJ of metabolizable energy and 11.50 % of crude protein per kg of DM.^10^ All animals were fed with alfalfa hay and corn silage (control) without or with raw garlic (GAR 0, 30, 50 and 70 g kg^-1^ of DM) on top of concentrate, (Table 1). Diets were adjusted weekly to account for changes in body weight and physiological status. Raw garlic was purchased from a local market in Hamadan county at the peak of their maturity. Meat grinder was used for fine grinding the fresh garlic bulb. The homogenized garlic mixed thoroughly with concentrate just before the morning feeding. This study was approved by the Institutional Animal Care and Use Committee of Urmia University, Urmia, Iran.

**Feed intake, apparent total-tract digestibility and body weight. **Diets were offered in equal amounts twice daily (08:00 hr and 16:00 hr). Feed consumption was recorded daily by weighing feeds offered to and refused by the goats. Samples of feed and orts were collected daily and kept frozen. On day 10 of each experimental period, goats were transferred to metabolic cages allowing the collection of feces for digestibility studies. For four consecutive days, feces were weighed and mixed daily, and a representative sample was taken and stored at −20 ˚C for later chemical analysis. Body weight was determined at the beginning and the end of each experimental period on two consecutive sampling days. 

**Table 1:** Ingredients and chemical composition of experimental diets for pre-parturition goats

**Item**	**Diet**
***Ingredient (g kg*** ^-1^ *** DM)***	
**Lucerne hay**	415.00
**Corn silage**	600.00
**Barley**	85.00
**Wheat bran**	200.00
**Vitamin and inorganic premix** 1	30.00
***Chemical composition ***	
**Dry matter** ** (%)**	91.50
**Crude protein** ** (%)**	11.50
**Metabolizable protein (%)**	9.30
**Effective rumen degradable protein (%)**	10.60
**Metabolizable energy** ** (MJ Kg** ^-1^ ** of DM)**	16.10
**Fermentable metabolizable energy** ** (MJ Kg** ^-1^ ** of DM)**	11.80
**Ether extract** ** (%) **	1.510
**Neutral detergent fiber** ** (%)**	37.74
**Acid detergent fiber** ** (%)**	20.38
**Calcium (%) **	0.58
**Phosphorus (%) **	0.28

1 Vitamin and mineral mix contained (per kg of DM): 7 mg of Co, 167 mg of Cu, 33 mg of I, 2660 mg of Mn, 27 mg of Se, 4660 mg of Zn, 1000 kIU of vitamin A, 200 kIU of vitamin D3, and 1330 mg of vitamin E, 67 g of NaCl, 33 g of S, 300 g of MgO, 200 g of mono calcium phosphate, and 420 g of limestone.

**Sample Collection and Analytical Procedures. **Analytical dry matter (DM) contents of basal diet, garlic, orts, and feces were determined by oven-drying at 105 ˚C for 48 hr.^11^ Ash contents of basal diet, garlic, orts, and feces were determined by incineration at 550 ˚C overnight, and the organic matter (OM) content was calculated as the difference between 100 and the percentage of ash.^11^ Crude protein (CP) was calculated as nitrogen (N) × 6.25. The concentrations of neutral detergent fiber (NDF) in basal diet, garlic, orts, and feces were determined as described by Van Soest *et al*.^12^ without the use of sodium sulfite. The acid detergent fiber (ADF) content of diet, garlic, orts, and feces were determined according to AOAC .^11^

Blood samples were collected from the jugular vein on day 13 and 14 of each experimental period at three hours after morning feedings. Blood samples were left at room temperature until clotting. Tubes were then centrifuged for 20 min at 2500 *g*. Serum for determination of glucose, NEFA, BHBA, total triglyceride, cholesterol, total protein, albumin, sodium, potassium, calcium and phosphorus were stored at −20 ˚C until analyses.

Serum concentration of β-Hydroxybutyrate (BHB) and non-esterified fatty acids (NEFA) were determined by D-3-hydroxybutyrate kit and NEFA Kit (Randox, Ardmore, UK), respectively. The concentration of plasma glucose, cholesterol, triglyceride, total protein, albumin, calcium and inorganic phosphorus were determined by auto-analyzer spectrophotometer (Model S-2100; Unico, New Jersey, USA) using commercial kits (Parsazmoon, Tehran, Iran). Serum concentrations of sodium and potassium were measured with flame photometery (Model PFP7; Jeneway, Essex, UK).

**Statistical analysis. **All data were analyzed as 4×4 Latin square using the PROC of GLM of SAS (Version 8.01; SAS Institute, Inc., Cary, USA). Source of variation in the model were goat, period, and treatment. Tukey’s multiple comparisons tests were used to determine differences between treatments. Differences were considered significant at *p *≤ 0.05. The following model was used for statistical analysis:

*Yijk = m + Gi + Pj + Tk + Eijk*

where, *Yijk* is the observed response, *m* is the overall mean, *Gi* is the effect of goat i, *Pj* is the effect of period j, *Tk* is the effect of treatment k, and *Eijk* is the residual error.

## Results

**Feed intake, total tract digestibility and body weight changes.** Table 1 and Table 2 respectively presents the results obtained from analysis of the chemical composition of basal diet and garlic fed to experimental animals. Dry matter intake (DMI) and body weight of treatment groups did not differ statistically from those of the control group, (Table 3). Apparent total tract digestibility of the OM, CP, ADF and NDF also were not significantly affected by the treatments, (Table 3).

**Table 2:** Chemical composition of the raw garlic

**Item**	**Percentage**
**Dry matter **	37.03
**Organic matter **	95.74
**Crude protein **	9.62
**Ether extract **	0.69
**aNDF **	6.70
**Acid detergent fiber **	5.21
**Calcium **	0.03
**Phosphorus **	0.02

aNDF = Neutral detergent fiber assayed with a heat stable alpha amylase expressed inclusive of residual ash.

**Blood metabolites. **Blood metabolites concentrations are shown in Table 4. The concentration of glucose was significantly increased by GAR70 compared to the other treatments. Also, the concentration of cholesterol in goats received 70 g kg^-1^ DM of garlic was lower (*p* < 0.05) than that of control group. There were no differences between treatments in serum BHB and NEFA concentrations (Table 4). However, garlic treated goats numerically had lower values of serum NEFA than those fed with the basal diet (0.30 versus 0.24 mmol L^-1^). No significant effects were found in triglycerides, total protein, albumin, calcium, inorganic phosphorus, sodium and potassium between groups, (Table 4). 

**Table 3:** Effects of raw garlic supplementation on dry matter intake, total apparent digestibility of nutrients and body weight changes in Mahabadi goats. Results are expressed as mean ± standard error (SE) of the means

**Parameters**	**GAR0**	**GAR30**	**GAR50**	**GAR70**	**SE**	***p*** ** value**
**Body weight (kg)**	59.82	60.00	59.73	59.90	0.57	Not significant
**Feed intake (kg DM per day)**	1.63	1.45	1.76	1.78	0.06	Not significant
***Apparent digestibility ***						
**Organic matter ** **(%)**	59.82	60.00	59.73	59.90	0.57	Not significant
**Crude protein ** **(%)**	60.42	59.88	61.30	61.27	0.44	Not significant
**Neutral detergent fiber** ** (%)**	55.32	56.12	57.41	56.23	0.81	Not significant
**Acid detergent fiber** **(%)**	43.26	43.31	44.11	43.37	0.73	Not significant

**Table 4:** Blood serum constituents of pre-parturient goats fed on rations supplemented with raw garlic. Results are expressed as mean ± standard error (SE) of the means

**Parameters**	**GAR0**	**GAR30**	**GAR50**	**GAR70**	**SE**	***p*** ** value**
**Serum glucose (mg dL** ^-1^ **)**	64.87^b^	65.25^b^	71.50^b^	84.12^a^	6.62	
**β-hydroxybutyrate** ** (mmol L** ^-1^ **)**	0.51	0.50	0.49	0.49	0.09	Not significant
**Non-esterified fatty acids (mmol L** ^-1^ **)**	0.30	0.28	0.27	0.24	0.14	Not significant
**Total triglycerides (mg dL** ^-1^ **)**	27.75	26.00	29.37	26.00	9.07	Not significant
**Cholesterol (mg dL** ^-1^ **)**	65.00^a^	63.75^ab^	64.00^ab^	61.88^b^	1.97	
**Total protein (g dL** ^-1^ **)**	6.42	6.37	6.30	6.75	1.43	Not significant
**Albumin (g dL** ^-1^ **)**	4.11	4.10	4.07	4.03	0.99	Not significant
**Calcium (mg dL** ^-1^ **)**	12.95	12.72	12.52	13.21	0.74	Not significant
**Phosphorous (mg dL** ^-1^ **)**	9.88	9.84	9.98	10.17	1.21	Not significant
**Sodium (mEq L** ^-1^ **)**	113.25	115	110.25	95.12	12.80	Not significant
**Potassium (mEq L** ^-1^ **)**	2.74	2.66	2.81	2.35	0.52	Not significant

* indicates significant differences at *p < *0.05;

** indicates significant differences at *p < *0.01.

## Discussion

The DMI, OM and CP did not differ between treatments. Generally the pungent odor of garlic can decrease palat-ability of the ration. Nevertheless, garlic supplementation at the selected levels did not affect the feed intake in this study, which was in accordance with previous reports with total mixed ration in lambs and cows.^13^^,^^14^

Plasma glucose concentration was significantly greater for animals supplemented with 70 g of garlic per kg of DM than the other treatments and numerically was higher for GAR50 and GAR30 than basal diet. This indicates that garlic constituents might influence the responses to glucose metabolism in pre-partum goats. These results are in agreement with those of other workers who fed garlic to the sheep and early lactation goats.^15^^,^^16^ However, these responses are in contrast with the finding of Chaves *el al. *who reported no difference in serum glucose concentration of growing lambs fed with diets supplemented with garlic compared with control ration.^13^ Furthermore, hypoglycemic effect of garlic in glucose-loaded diabetic rats illustrated previously which might be due to the inhibition of glucose absorption from the intestine and/or the enhancement of glucose utilization by increasing the pancreatic secretion of insulin from existing b-cells.^17^^,^^18^ In this study the increased plasma glucose concentration might be due to the enhancement of gluconeogenesis process by garlic supplemented diet. As reported previously, ruminal propionate is considered the most important single precursor of glucose when its availability is high.^19^ Garlic appear to have great potential for improving of propionate to acetate ratio without affecting nutrient utilization.^8^^,^^9^ This may explain the higher blood concentration of glucose in the current study. Serum glucose improvement in parallel with ruminal propionate has been illustrated previously by Kholif *et al*.^16^ in garlic oil treated lactating goats. 

Taking into account the available information about the possibility of using levels of certain blood constituents in the determination of energy status in the ruminant, it is postulated that levels of plasma NEFA are the most reliable criterion. Together with this, levels of blood ketones are also considered useful and hypoglycemia is also considered as an indication of low energy intake.^20^^-^21 The plasma concentrations of NEFA’s are useful indicator for monitoring the energy status of goats during the last month of gestation. Additionally, the availability of glucose prevents the formation of ketone bodies. Hence, the higher glucose value suggested that components of garlic might play role in reducing negative energy balance on animals fed garlic. As previously reported, garlic had a potential to alter rumen ecology and increase propionate synthesis in sheep and in dairy goats,^9^^,^^16^ which in turn might promote gluconeogenesis and insulin secretion.^22^ Therefore, the anti-lipolytic effect of insulin should decrease plasma NEFA and reduce hepatic conversion to triglycerides and ketones in garlic treated animals. In the current study serum concentration of total triglycerides, NEFA and BHB did not differ among the treatment. However blood NEFA concentration numerically were lower in animals fed garlic supplemented diets as compared to those fed the basal diet. 

Total protein and albumin concentrations were not affected by dietary treatments at pre-parturition period. This is in agreement with the results of some reports in finishing pigs,^23^^,^^24^ however, is in contrast with Kholif *et al*. who found higher serum albumin and total protein values in garlic oil supplemented lactating goats.^16^ This discrepancy might be explained by differences in the experimental procedure kind of animals, physiological status and various garlic compounds used.

The hypolipidemic effect (triglycerides and cholesterol lowering properties) of garlic has been proved in previous studies in humans^25^, rats^26^ as well as in laying hens.^27^ This effect is probably due to the inhibition of enzymes involved in fatty acid and cholesterol synthesis.^17^ Similarly serum cholesterol level of goats fed garlic at 70 g kg^-1 ^of DM was significantly lower than the other treatments. However, the results of the present study showed that garlic supplementation had no influences on the concentrations of triglycerides in serum. These results were in agreement with the previous reports in lambs and dairy goats. ^13^^,^^28^ When energy balance is negative, animals always mobilize the lipids stored in adipose tissues, mainly in the form of NEFA. In the present study, the concentration of NEFA did not change with increasing garlic supplementation, suggesting that the mobilization was not elevated.

Mahusoon *et al*. reported marked breed differences in mineral metabolism in goats.^29^ To the best knowledge of the authors, there is no published report on the influence of garlic on metabolism of minerals and electrolytes in pre parturient goats. In the current study, garlic did not affect calcium, inorganic phosphorus, sodium and potassium content of blood.

In conclusion, feeding pre-partum goats with raw garlic increased serum glucose concentration compared to the un-supplemented control group, in this trial. The results of this study showed that garlic is a promising additive to influence positively the energy status of pre-partum goats. Increased serum glucose concentration could support the hypothesis that garlic supplementation can improve efficacy of feeding which in turn may improve production performance of the pregnant goats. Further researches are needed to determine the mechanism of garlic constituent action on feed efficiency and performance of ruminants.
